# Time to recovery and its predictors among children aged 6–59 months having uncomplicated severe acute malnutrition attending an outpatient therapeutic program in Northeast Ethiopia: prospective cohort study

**DOI:** 10.3389/fnut.2024.1407931

**Published:** 2024-08-07

**Authors:** Fentaw Wassie Feleke, Setamlak Adane Masresha, Getahun Fentaw Mulaw

**Affiliations:** Department of Public Health, School of Public Health, College of Medicine and Health Sciences, Woldia University, Woldia, Ethiopia

**Keywords:** GMP, OTP, prospective cohort study, SAM, time to recovery

## Abstract

**Introduction:**

There are insufficient data regarding the variables influencing recovery times, despite the accessible outpatient therapy program (OTP) bringing services for treating severe acute malnutrition (SAM) closer to the community. Therefore, this study aimed to identify the factors influencing the recovery duration in children with uncomplicated SAM between the ages of 6 and 59 months who were attending an OTP in North Wollo, northern Ethiopia.

**Methods:**

From February 2021 to July 2021, 356 children, ages 6–59 months, enrolled in a facility-based prospective cohort study. An interviewer administered a semi-structured questionnaire once a week to acquire anthropometric measures. The data were imported into Stata version 14.2 for analysis from EPI data entry version 4.6.06. The time to recovery for each attribute was determined using a log-rank test, a survival curve, and a Kaplan–Meier estimate of the median time to recovery. The Cox Proportional-Hazards Model was used to identify independent predictors of recovery time; statistical significance was indicated at 95% CI and a *p*-value of 0.05.

**Results:**

With a recovery rate of 74.7%, the median recovery period was 56 days. Frequency of growth monitoring and promotion (GMP) service utilization [AHR = 1.622 (95% CI: 1.052–2.130)], cough [AHR = 0.385 (95% CI: 0.176–0.843)], maternal delivery at health center [AHR = 1.448 (95% CI: 1.023-2.050)], and maternal literacy [AHR = 1.445 (95% CI: 1.019–2.058)] were determinants of time to recovery.

**Conclusion:**

The median recovery period was 56 days with a recovery rate of 74.7%. Regular utilization of GMP services, maternal delivery at the health center, and cough at admission were independent predictors for this study. As a result, there should be a greater emphasis on the importance of girls’ (future mothers’) education and nutrition counseling, particularly the integration of GMP service components into institutional delivery/for girls/women who have received little education on how to improve time to recovery and the success of the OTP.

## Introduction

Eating and drinking anything you want as long as it meets the body’s major nutritional needs in a sufficient and balanced manner to prevent sickness and improve quality of life, or consumption of essential nutrients on purpose to prevent hunger from becoming an emotion, is considered adequate nutrition (1, 2). The cornerstone of healthy physical and cognitive development in children is nutrition, which has long-term effects on people’s health and entire civilization (3).

Malnutrition is a leading cause of morbidity, death, and long-term developmental deficiencies that are extremely preventable. It causes devastation generation after generation and leaves a dreadful legacy for the next, beginning in early childhood and lasting into old life (2). Severe wasting (weight for height less than the World Health Organization’s (WHO) reference z score), mid-upper arm circumferences (MUAC) less than 11.5 cm, and evidence of nutritional pitting edema are the criteria for severe malnutrition. Given that it is a potentially fatal illness, immediate treatment is needed (4, 5). SAM is still an issue that affects the entire world, persisting at unacceptably high rates and endangering the lives of millions of children (6). Even with small improvements in a few nutrition indicators, the current rate of advancement will not allow the world to meet its 2025 nutrition targets.

Globally, approximately 45.5 million children were wasted, and 13.6 million were severely wasted, with more than three-quarters from Asia, and 22% from Africa (7). Compared to the global goal of reducing waste to less than 5%, the rate of waste is alarming, compounded by natural and man-made disasters (8–11). Ethiopia has been hit by a series of natural and man-made calamities, most notably the cyclic drought, which has had a severe impact on the country’s efforts to reduce acute malnutrition. Although wasting prevalence decreased from 13 to 11% between 2016 and 2023, the weight of SAM in the country is rapidly increasing because of the present civil war, rising food prices, and other factors. In the research region of Amhara state, the prevalence of wasting is 15% (12, 13). As a result, acute malnutrition accounts for a total loss of $230 million and up to 64% of the economic cost (14).

Community Management of Acute Malnutrition (CMAM) is effective in promoting the speedy recovery of SAM children and preventing repeat crises. However, the coverage remains at 71.1%, as reported in 2022, and a large number of SAM children are being missed (11).

The OTP provided more coverage (79%–95% of SAM children) for children suffering from uncomplicated SAM to recover, while treatment success is limited by various aspects that need to be addressed (15).

A study discovered that SAM children had a high recovery rate of 92%, a low death rate of 0.1%, and tolerable default rates (7.5%). The implementation of CMAM has increased access to malnutrition care close to home while significantly lowering deaths. However, only approximately 20% of children with SAM receive treatment in a facility. The OTP makes it easy to treat children at home, with maximum coverage (16). To improve coverage and treatment success, the program emphasizes SAM children over the age of 6 months who do not have any medical concerns or have been transferred out of inpatient care. Therapy outcomes are defined as cured, defaulted, dead, or non-respondent to therapy, and they serve as indicators of therapeutic success (5, 17).

The SPHERE standard sets acceptable levels of mortality, recovery rate, and default rate as less than 10%, greater than 75%, and less than 15%, respectively (18).

Previous research has focused on inpatient therapy, but there are inadequate data to identify factors that may affect time to recovery, treatment success, and outcomes in prospective cohort study design. Furthermore, the OTP targets 80% of SAM children, therefore evaluating program success is critical for policy decisions (5, 17).

North Wollo has severe dietary issues, which are exacerbated by inadequate agricultural productivity. However, there is a lack of research into the factors that influence how long it takes people to recover from SAM, which is critical in dealing with the current situation. This study sought to close this knowledge gap by presenting important evidence unique to the context of North Wollo, where SAM is common, but evidence is scarce. Therefore, this study aimed to determine the time to recovery and its predictors among children aged 6–59 months having uncomplicated severe acute malnutrition attending an outpatient therapeutic program in Northeast Ethiopia.

## Materials and methods

### Study setting, design, and area

The study used a facility-based prospective cohort study design and ran from Febrauary 20 /2021–July 20/ 2021. The North Wollo Zone is situated 251.75 km from the regional state’s seat, Bahir Dar, and 520 km from Addis Ababa. North Wollo is one of 11 zones of the Amhara Region of northern Ethiopia. It is bordered on the south by South Wollo, west by South Gondar, north by Wag Hemra, northeast by Tigray Region, and east by Afar Region. It is subdivided into 11 Woredas and 312 kebeles (276 rural and 36 urban). Cereals are the staple foods. Most of this zone is mountainous and characterized by steep slopes, which are unsuitable for agriculture and severely limit the cultivated area.

According to the Central Statistical Agency of Ethiopia (CSA) 2007 Census projection, a zone with an area of 12,172.50 km2 has a total population of 1,788,901, of whom 752,895 are men (895,189) and 747,408 women (893, 712). Approximately 241,502 of these are 6- to 59-month-old children. The zone’s governmental health facility has 306 health posts, 65 health centers, 5 primary district hospitals, 1 referral hospital, and 5 health centers.

### Population

The source population included all SAM children between the ages of 6 and 59 months who were admitted to OTP in North Wollo Zone for treatment. The study population comprised children aged 6–59 months who were screened since January 20 and admitted for treatment in the approved OTP sites in North Wollo Zone. The study unit was composed of SAM children who stayed for the recommended amount of time and had a variety of outcomes.

### Inclusion and exclusion criteria

#### Inclusion criteria

The mid-upper arm circumference (MUAC) of less than 11.5 cm, bilateral pitting edema rating of mild (+) or moderate (++), absence of medical complications, and successful completion of the appetite test in children aged 6 to 59 months were the inclusion criteria for SAM.

#### Exclusion criteria

Situations involving disability for which an anthropometric assessment is unsuitable.

### Study variables

#### Outcome variable

➢ Time to recovery

#### Independent variables

✓ Features of the child (age, weight, MUAC, admission criteria, cough, and diarrhea)
✓ Household factors (wealth index, food security, paternal occupation, head of the household, number of children under 5 years, size of the family, paternal education level, and media exposure)
✓ Maternal factors (age, parity, occupation, education level, ethnicity, religion, and marital status)
✓ Nutrition and health-related factors (distances between the child’s residence and the OTP sites, antibiotics, nutrition counseling, frequency of meals, variety of diets, minimum acceptable diet, GMP, vaccination against measles, and deworming).

#### Operational definitions

1. Admission criteria: For uncomplicated SAM to be treated at OTP, the following criteria had to be met for admission: MUAC of less than 11.5 cm, bilateral pitting nutritional edema ratings of mild (+) or moderate (++), no coexisting medical complications, and children between the age of 6 and 59 months having completed the appetite test (17).
2. Average length of stay: The length of a patient’s treatment, calculated just for those who have recovered, and counted from the moment of admission to discharge (4, 5).
3. Censored: Events included children who defaulted, did not respond, passed away, and transferred to inpatient facilities or other OTP sites outside of the research area (19).
4. Critical times for handwashing: This includes before preparing food, before eating a meal, before feeding a child, after visiting a latrine, after cleaning the child’s bottom or disposing of the child’s stool, and after cleaning a house or the environment (20).
5. Death: Referred to the patient who died while he/she was in the OTP until the end of the study (4, 5).
6. Defaulter: Missing two straight appointments and verified as alive by a home visit (4, 5).
7. Event: The recovery of children who were severely malnourished throughout their enrollment in the program and/or after the study (19).
8. Food security: It was divided into two categories by the HFIAS tool: food insecure if the head of the household answered “yes” to at least one of each item on the list of nine, and food secured if the household respondents answered “no” to all of the items (21).
9. Good maternal handwashing practice: It occurs when mothers who have given birth or care for a child score at least three of five handwashing practice questions (22, 23).
10. Growth monitoring (GM) and promotion attendance/frequency (GMP): Child weighing must get GMP services at least five times a year, four, and three times a year for ages 6 to 11, 12 to 23, and 24 to 59 months, respectively (24–26).
11. GMP service utilization: Measured when the respondents met the requirements, which included having the GM card available, regularly weighing, receiving dietary advice from medical professionals, being able to read the GM chart graph, and being knowledgeable about the benefits of regular weighing at the time of data collection. Those who did not meet these requirements were deemed improperly using the GMP service (24, 25).
12. Handwashing: The act of cleaning hands with water and soap to remove dirt, contaminants, and microbes.
13. Handwashing practice: It is routinely/through the life/action of washing surfaces of lathered hands, followed by rinsing under a stream of water with soap in the critical time before preparing food, before eating food, after eating food, after cleaning the baby’s bottom, after disposing of child feces, and after visiting the toilet (22).
14. Medical complications: Children with any of the following severe medical complications should receive hospital-based medical management and cases should be admitted swiftly for inpatient treatment (5, 27) such as poor appetite, intractable vomiting, convulsion, lethargy, alertness, unconsciousness, hypoglycemia, high fever (axillary temperature ≥ 38.50.C), hypothermia, dehydration, lower respiratory tract infection (e.g., pneumonia), severe anemia, persistent diarrhea, eye sign of vitamin A deficiency, and severe skin lesions (e.g., severe dermatosis) (4).
15. MUAC gain (cm): Was the difference between the MUAC recorded at discharge and the MUAC at admission (28–30)?
16. New admissions: New admissions were those who were admitted directly to OTP to get started on the nutritional treatment (5).
17. Non-responder: Referred to the patient who did not reach the discharge criteria until ≤112 days (5).
18. Re-admission: Re-admitted for treatment in OTP due to relapse or a returned defaulter who meets the admission criteria (4, 5).
19. Relapses: Cured within the past 3 months and currently meets the admission criteria for OTP (4, 5).
20. Recovered: Children whose weight for height/length Z-score is ≥ −2 SD, and the child has had no edema for at least 2 weeks, or whose MUAC is ≥12.5 cm and the child has had no edema for at least 2 weeks. No edema for 2 consecutive weeks and clinically well and alert (5).
21. Successful treatment outcome: It is an outcome of children under OTP treatment, which is recovered, and discharged within ≤112 days of OTP intervention (5).
22. Time to recovery: Refers to the number of days that the patient stays in the OTP until cured or discharged (5).
23. Transfer to another outpatient service: A child with SAM in treatment at one site moves to another outpatient site to continue treatment (5).
24. Treatment outcome of OTP: A result of children receiving OTP, which included recovery, defaulters, non-responders, and mortality (5).
25. Unsuccessful treatment outcome: An outcome of children under OTP treatment who defaulted, were non-responders, or were transferred out to inpatient treatment (5).
26. Weight gain: Refers to the increase in weight of the patient after being admitted to the OTP (25, 30).

### Sample size determination and sampling technique/procedures

#### Sample size determination

##### Sample size determination for treatment outcomes of OTP

The minimum sample size required for OTP treatment outcome was estimated using a single population proportion formula *n* = $\frac{{\left(Z\alpha /2\right)}^{2}*P\left(1-P\right)}{d^{2}}$ = 210 with the following assumptions:

*p* = 93.4% (the recovery rate of children treated under OTP) (31).

*n* = minimum sample size, Zα/_2_ = Z value for 95% confidence level = 1.96, d margin of error (5%), none response allowance (10%), and design effect 2.

The sample size was computed using STATA (version 14) through the following statistical assumptions: considered two-sided significant level (α) of 5%, power 80%, Zα2 value at 95% confidence interval = 1.96 [recovery rate = 62.13%, and hazard ratio (HR) = 0.66] (32, 33).


$$e=\frac{\left(Za/2+ZB\right)2}{{\left(\text{lnHR}\right)}^{2}p\left(1-p\right)}=>\frac{\left(1.96+0.84\right)2}{{\left(0.66\right)}^{2}\;0.6213\left(1-0.6213\right)}=>186.67$$

*n* = $\frac{e}{p\left(e\right)}$ = 186.67/0.6213 = 300 was the sample size. Finally, a 10% contingency was added for a non-response rate which was 330 (33) (Table 1).

**Table 1:** Sample size calculation by using MedCalc software using survival analysis (log-rank test) after searching different related literature for recovery rate among SAM children aged 6–59 months treated at OTP with the assumption of type I error 0.05 and type II error 0.20 (34).

Variables	Cured	Censored	% outcome	AHR	(10%) none response allowance	DE	Total sample size
Distance from OTP site (35)							
<2 h	141	3	95.6	2.91	132	2	264
≥2 h	97	45	77.4	1			
Vitamin A supplementation (35)							
No	151	44	68.3	0.39	66	2	132
Yes	87	4	97.9	1			
Vomiting at admission (36)							
Yes	10	2	12.2	0.43	39	2	78
No	244	133	60.0	1			

AHR, adjusted hazard ratio; DE, design effect.

Of the above four options, the final required sample size was taken as 356, as it was the largest of all calculated sample sizes taken from the prospective follow-up study (Table 2).

**Table 2:** Sample size calculation for determinants of time to recovery using Open-Source Epidemiologic Statistics for Public Health (Open Epi) Version 3.01 (https://www.openepi.com/SampleSize/SSCohort.htm), considering the following assumptions:

Variables	CI (%)	Power (%)	Ratio	(%) outcome in unexposed	(%) outcome exposed	10% allowance	DE	Minimum final sample size
Distance (35)	95	80	1	68.3	97.9	62	2	124
Amoxicillin (36)	95	80	1	58.7	79.2	178	2	356

CI, Confidence interval; DE, design effect.

### Sampling technique and procedure

**Figure 1:**
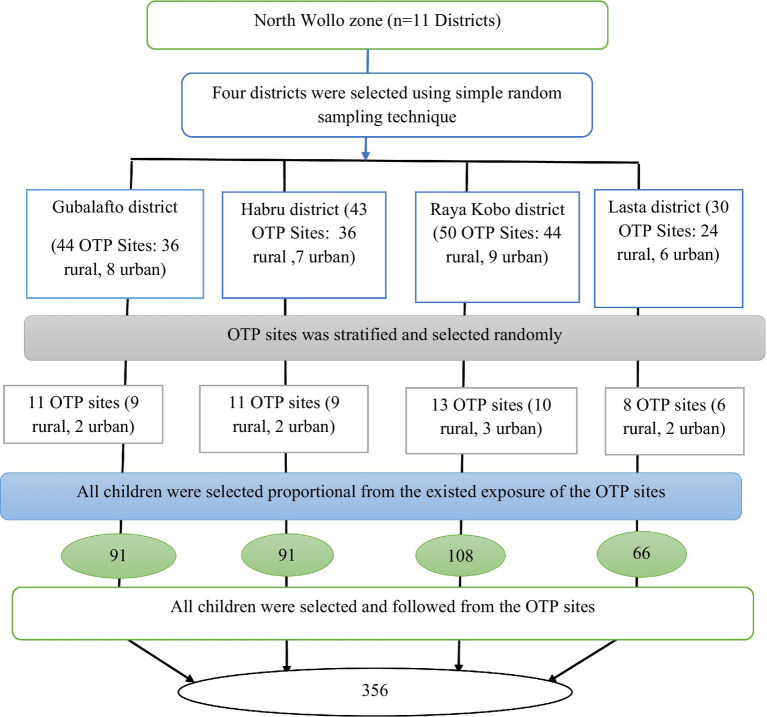
Sampling methods and procedures for time to recovery and its predictors among children aged 6–59 months having uncomplicated SAM attending an OTP in North Wollo, Northeastern Ethiopia, 2021.

A multi-stage sampling method was used. It was assumed that the population was homogeneous among the functional OTP sites. Finally, a total sample size of 356 was distributed to the selected OTP sites for their new SAM cases based on population proportional to size (Figure 1).

### Data collection method, tools and personnel, and data quality assurance

Information was collected using a semi-structured questionnaire. Most of the questions were adapted from relevant literature (12, 14, 15, 18, 28, 29). The questionnaires were written in English, translated into the local language (Amharic), and then back into English, and their consistency was reviewed by other language experts. Before the actual study began, the primary investigator provided 3 days of training on equipment, techniques, variables on the data collection sheet, ethical issues, and how to proceed with OTP follow-up data collecting for this project. Training included MUAC measurements, weight for height measurement, and edema assessment. Following, a pretest was conducted on 10% of the sample size outside of the research locations.

The principal investigator (PI) and co-investigators also provided tight supervision during baseline data collection and follow-up. Every week, co-investigators and the PI reviewed the completed data form for consistency and completeness of information.

Baseline data on socio-demographics, HFIAS questionnaire, clinical symptoms, and individual dietary diversity were collected at the home level by trained professionals of each health extension program nutritional screening routine activity. Following the initial assessment, follow-ups were planned every week. Children were examined using the same measuring devices at their scheduled follow-up appointments.

Anthropometric measurement equipment was calibrated using standard techniques. Before use, the scale was calibrated with a known 1 kg weight, and the scale was reset to zero for each child. The hanging Salter scale and the Uni-scale for older children were used to determine the weight of the children to the closest 0.1 kg. The MUAC tape was calibrated by measuring each tape with a 20-cm plastic pipe. The MUAC was measured on the left arm at the midpoint between the tip of the shoulder (olecranon) and the tip of the elbow (acromion) to the nearest 0.1 cm using MUAC tapes and recorded immediately after measurement. During the nutritional edema determination, normal thumb pressure was administered to both feet for at least 3 s; if a shallow print persists on both feet, the child has edema. Only children with bilateral edema were found to have nutritional edema.

### Data processing and analysis

The data were entered into Epi data entry version 4.6.06. The data were exported, cleaned, and analyzed using SPSS version 25 and STATA 14.2. Descriptive statistics was reported using frequencies, percentages, median, and texts. Proportions of the OTP outcomes were reported as recovered, non-respondent, defaulter, transferred out, and death using frequency and percentages.

The wealth index was computed using principal component analysis as an indicator of household wealth. The median time to recovery was determined using the Kaplan–Meier vs. predicted survival analysis. The length of stay was transformed into survival time data by subtracting the date of the first supplement from the date of recovered/discharge/censored (end of study date).

Predictors of time to recovery were identified using a multivariable Cox Proportional-Hazards Model. All independent variables that were nearly associated with the dependent variable at *p*-value < 0.25 during bivariate analysis were considered candidates for the multivariable model. The assumption of the Cox Proportional-Hazards Model was assessed. It means that the explanatory variable only changes the chance of failure not the timing of periods of high hazard. The explanatory variable acts directly on the baseline hazard function and not on the failure time and remains constant over time. If the two lines or curves were parallel, then the hazards can be considered proportional. The vertical separation of the two lines gave an appropriate estimate of the log hazard ratio. Finally, the Cox Proportional-Hazards Model analysis was done adjusted hazard ratios with the 95% CI were estimated, and a *p*-value < 0.05 was used to declare the presence of a significant association between dependent and independent variables.

## Results

### Socio-demographic/economic characteristics

A cohort of 352 children were followed prospectively with a response rate of 98.8%. The mean age of mothers having children aged 6–59 months was found to be 32 ± 6 years. Three-fourths of maternal 257 (73%) religion were Orthodox, and the rest were 89 (25%) Muslim and 6 (2%) protestants. Approximately 311 (88.4%) were Amhara and the rest were Tigray in ethnicity. One in three mothers was found in a poor economic status household (Table 3).

**Table 3:** Socio-demographic characteristics of study participants in North Wollo Zone, Amhara regional state, Ethiopia 2023.

Variable	Categories	Frequency	%
Maternal marital status	Single	17	4.8
	Married	307	87.2
	Divorced	18	5.1
	Widowed	10	2.8
Maternal education status	Illiterate	127	36.1
	Read and write	105	29.8
	Formal education	120	34.1
Paternal education status	Illiterate	107	30.4
	Read and write	131	37.2
	Formal education	114	32.4
Household wealth status	Poor	117	33.2
	Medium	158	44.9
	High	77	21.9
Maternal occupation	Housewife	268	76.1
	Merchant	10	2.8
	Civil servant	22	6.3
	Daily laborer	34	9.7
	Farmer	11	3.1
	Student	7	2.0
Household resource controller	Mother	175	49.7
	Father	141	40.1
	Someone else	36	10.2

### Maternal healthcare-related characteristics

Half of 178 (50.6%) of study participants were female. Three-fourths of 269 (76.4%) of them were from rural areas. The majority 281 (79.8%) of children were aged between 12 and 23 months. One in four 88 (25%) of the study participants were found within food-secured household family. Approximately 287 (81.5%) and 288 (81.8%) of children had taken amoxicillin and vitamin A supplementation, respectively. There were 266 (75.6%) mothers/ caregivers who travelled less than 2 hours to obtain OTP. One in every four mothers/caretakers has sold and purchased RUTF (Tables 4–6).

**Table 4:** Maternal healthcare and water sanitation and hygiene-related characteristics during OTP services in North Wollo Zone, Amhara regional state, Ethiopia 2023.

Variable	Categories	Frequency	%
ANC follow-up	No	121	34.4
	1–3	144	40.9
	4 and above	87	24.7
Place of delivery	Home	149	42.3
	Health center	142	40.3
	Government hospital	61	17.3
PNC follow-up	No	114	32.4
	1–2	201	57.1
	3 and above	37	10.5
Nutritional counseling during ANC and PNC	No	106	30.1
	Yes	231	65.6
	Do not remember	15	4.3
Currently family planning utilization	Yes	250	71.0
Currently used family planning type	Pills	5	1.4
	Injectable (Depo)	210	59.7
	Norplant	35	9.9
Maternal nutritional status	MUAC below 23 cm	87	24.7
	MUAC 23 and above	265	75.3
Iron folic acid supplementation	90 and above	212	60.2
Maternal handwashing practice at critical times	Good	163	46.3
	Poor	189	53.7

**Table 5:** Child healthcare-related characteristics in the OTP in North Wollo Zone, Amhara regional state, Ethiopia 2023.

Variable	Categories	Frequency	%
Child age in completed months	6–11	50	14.2
	12–23	281	79.8
	24–59	21	6.0
Birth order	First	68	19.3
	Second	76	21.6
	Third and above	208	59.1
Children referred by OTP sites	Community Volunteer	28	8.0
	Health workers	27	7.7
	Self	297	84.4
Distance from OTP sites in hours	Below 2 h	266	75.6
	2 and above 2 h	86	24.4
General food ration	Yes	122	34.7
Diarrhea	Yes	64	18.2
Vomiting	Yes	78	22.2
Fever	Yes	76	21.6
Cough	Yes	77	21.9

**Table 6:** Routine medications and feeding practices for children aged 6–59 months in the OTP in North Wollo Zone, Amhara, Ethiopia 2023.

Variable	Categories	Frequency	%
Ever breastfeeding	Yes	352	100
Colostrum is given to a child	No	6	1.7
	Yes	282	80.1
	Do not remember	64	18.2
Pre-lacteal feeding	Yes	92	26.1
Starting age for complementary feeding	Below 6 months	84	23.9
	Above 6 months	52	14.8
	Exactly at 6 months	216	61.4
Family decision maker for OTP service	Mothers	48	13.6
	Fathers	61	17.3
	Both fathers and mothers	243	69.0
Admission criteria	Nutritional edematous	91	25.9
	MUSIC	261	74.1
Children screened by	HEW	262	74.4
	Health professional	90	25.6
Screening place	Health post	266	75.6
	Health center	54	15.3
	Village/home	32	9.1
MAM treatment history	Yes	91	25.9
SAM treatment history	Yes	80	22.7
Registration for general food ration	Yes	122	34.7
Amoxicillin	Yes	287	81.5
Measles (*n* = 334)	Yes	285	85.3
Ever vitamin A supplementation	Yes	288	81.8
Deworming (*n* = 21)	Yes	18	85.7
Malaria treatment	Yes	60	17.1
Ever health and nutrition counseling	Yes	265	75.3
GMP service utilization	Yes	185	52.6
Home visit	Yes	274	77.8
RUTF sharing	Yes	84	23.9
RUTF selling	Yes	91	25.9

### Child healthcare-related characteristics in the OTP services

Approximately 281 (79.8%) of the children were aged 12 to 23 months. Almost one out of every five children was born first or second. One out of every five children experienced diarrhea, vomiting, coughing, or fever (Table 5).

### Time to recovery and treatment outcomes of SAM children aged 6–59 months

**Figure 2:**
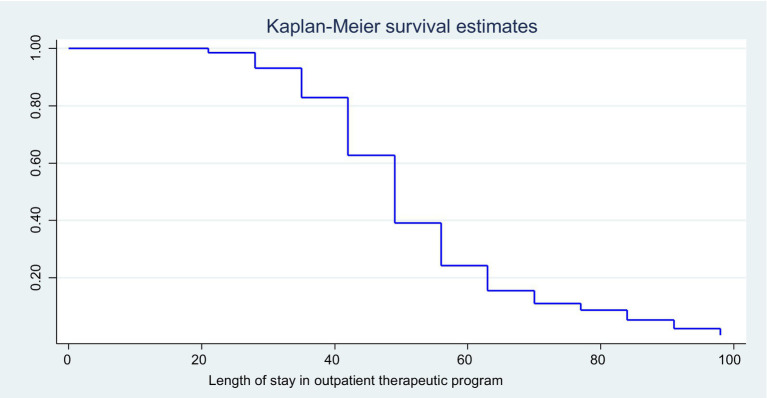
KM survival estimates of the enrolled children at the OTP in North Wollo Zone, Amhara regional state, Northeastern Ethiopia, 2023.

In this study, 263 (74.72%) had been diagnosed as marasmus, and 91 (25.85%) had been diagnosed as nutritional edematous. From the total recruited study subjects, 263 (74.72, 95% CI, 70.15–79.28) were successfully recovered. On the contrary, the rest of the children have not developed the event. The reasons for failure were defaulter and transfer out. The mean weight gain of the recovered children was 6.65 g/kg/day. The median time to recovery was 56 days (8 weeks) as determined by the Kaplan–Meier (KM) survival estimate (Figure 2).

### Comparison of time to recovery among the different groups (the KM survival curve)

The KM survival estimate was used to compare survival probability across predictor groups. In addition, the log-rank test was used to assess the significance of the difference in survival probability between predictors. As a result, survival time estimates for several factors such as cough, amoxicillin, sharing RUTF, selling RUTF, MAM treatment history, SAM treatment history, household food security status, and maternal nutrition status were substantially significant at 95% CI.

**Figure 3:**
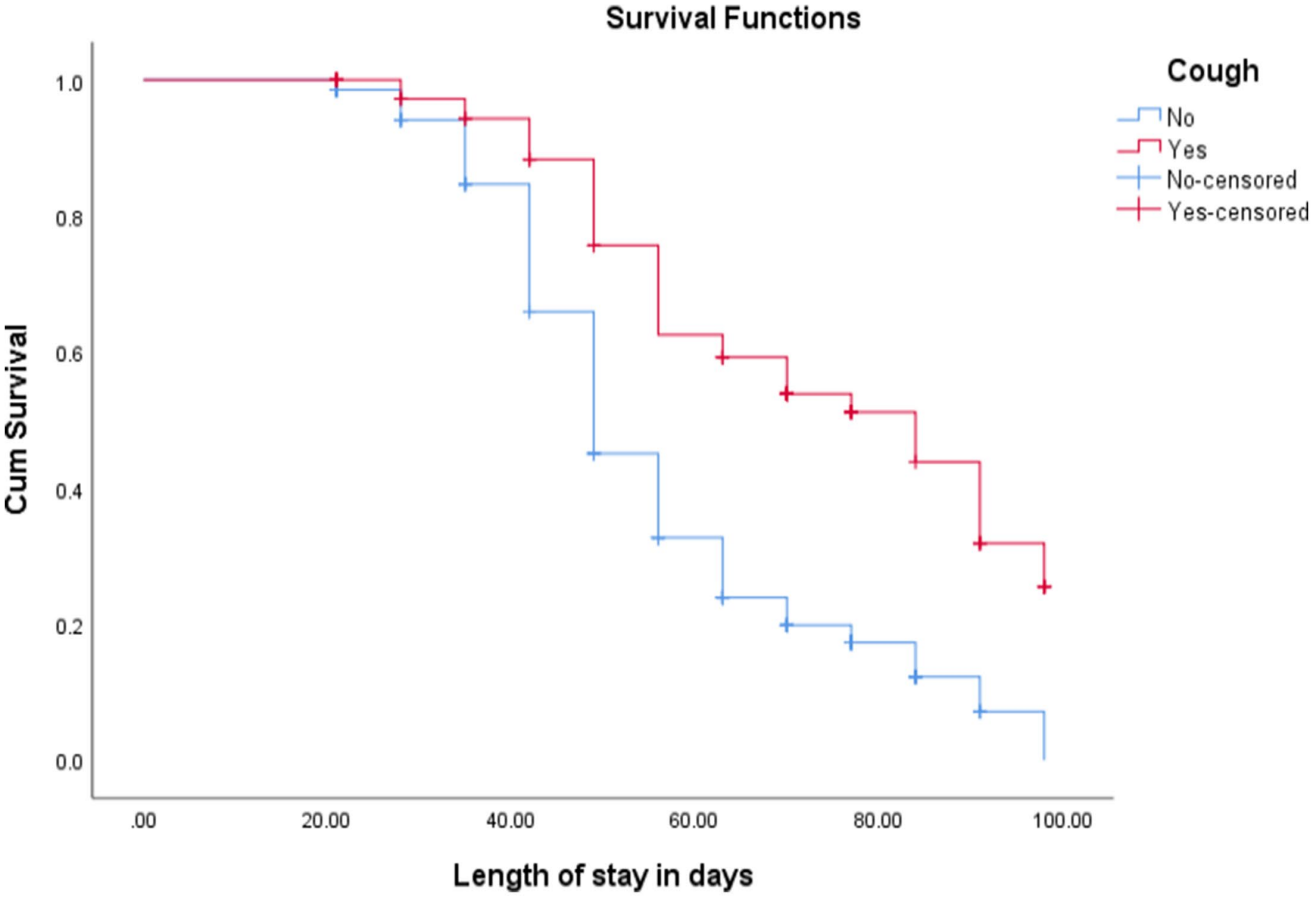
KM curve shows children with uncomplicated SAM admitted at OTP with cough and without cough in North Wollo Zone, Amhara regional state, Northeastern Ethiopia, 2023.

The KM survival curve for cough estimates children free from cough had better cure rates and short median length of stay. The cure rate and median length of stay for cough-free children were 227 (82.6%) and 49 ± 1 days, respectively, while children with a history of cough were 36 (46.8%) and 84 ± 10 days, respectively [(log-rank test =32.35, *p* < 0.001; Figure 3)].

**Figure 4:**
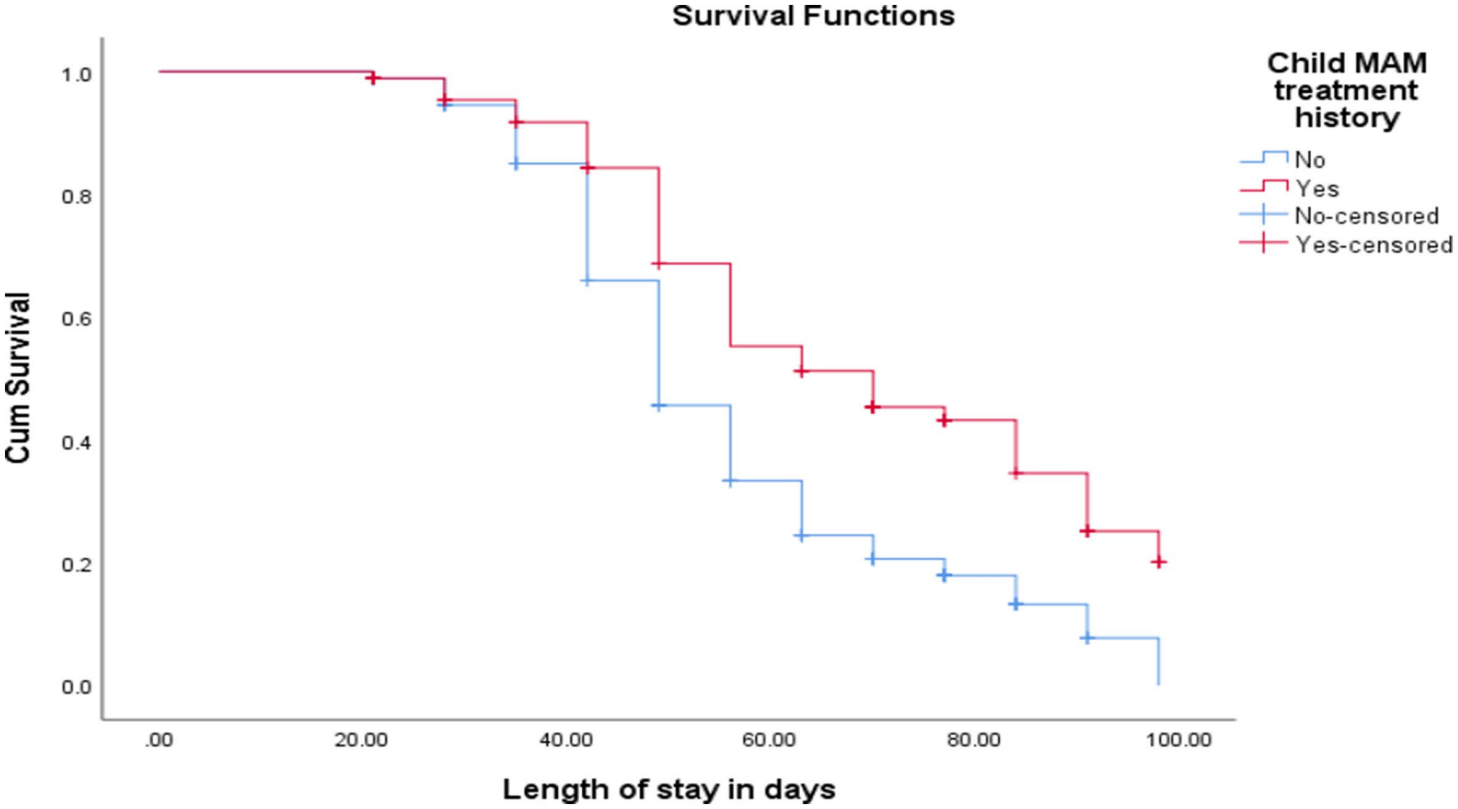
KM curve shows SAM children’s treatment history of MAM at admission at OTP North Wollo Zone, Amhara regional state, Northeastern Ethiopia, 2023.

The cure rate and median length of stay for children with MAM treatment history were 100% and 70 ± 10 days, respectively, while for children without a history of MAM treatment were 175 (66.3%) and 49 ± 1.4 days, respectively [(log-rank test = 21.8, *p* < 0.001; Figure 4)].

**Figure 5:**
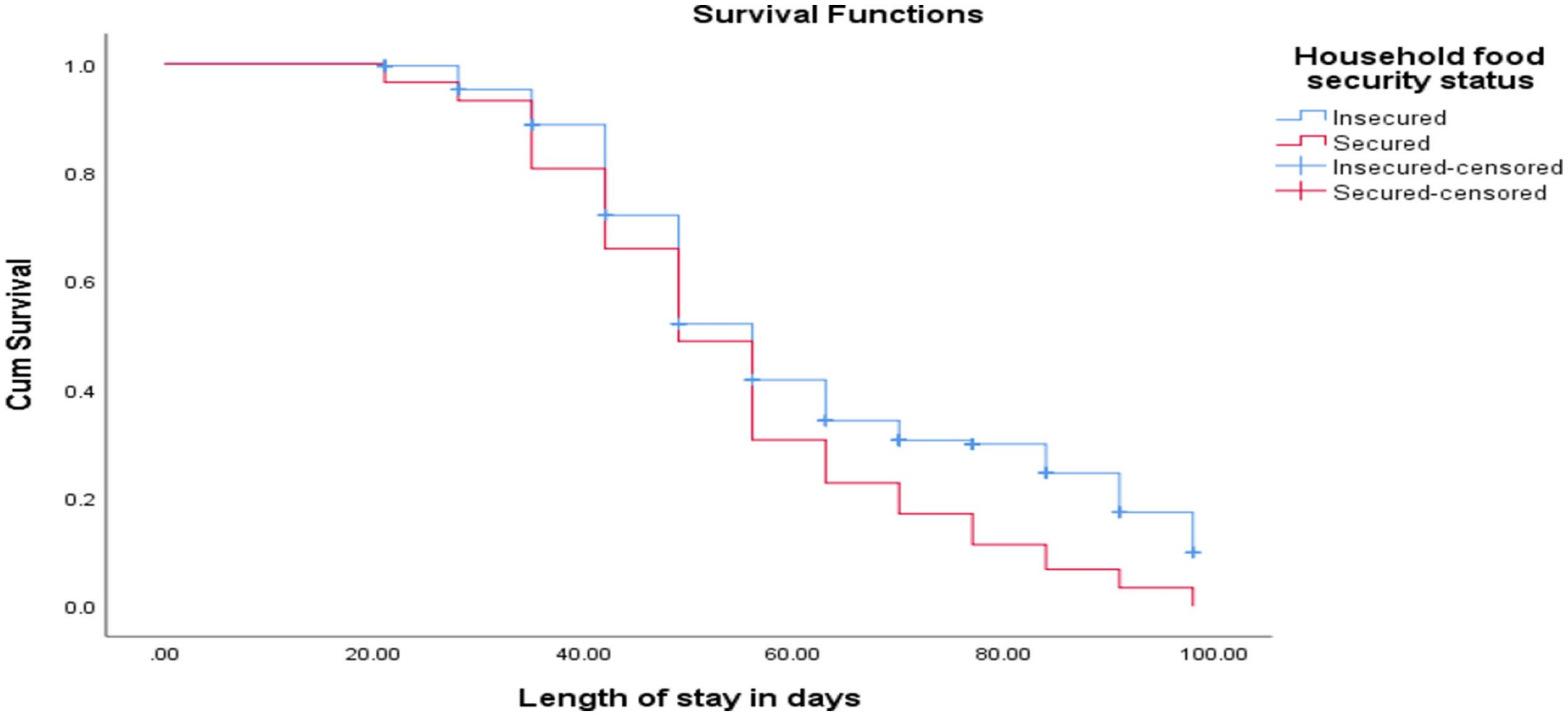
KM curve shows SAM children with food-secured and food-insecured households at OTP North Wollo Zone, Amhara regional state, Northeastern Ethiopia, 2023.

The cure rate and median length of stay for children of food-secured households were 88 (100%) and 49 ± 2 days, respectively, while children with a history of food insecurity were 175 (66.3%) and 56 ± 1.5 days, respectively [(log-rank test = 11.0, *p* < 0.001; as shown in Figure 5)].

**Figure 6:**
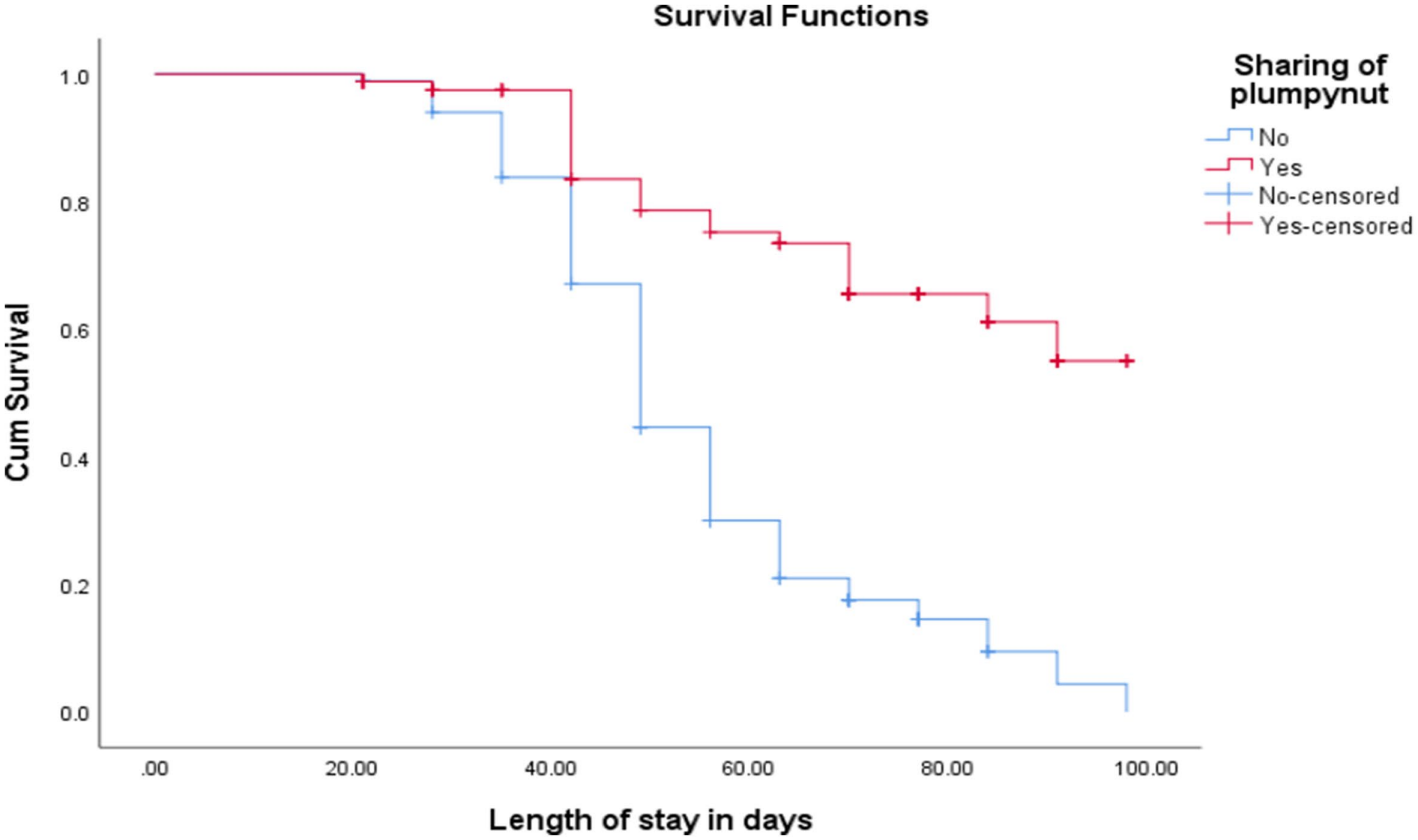
Comparison of survival curve for children with sharing RUTF at OTP North Wollo Zone, Amhara regional state, Northeastern Ethiopia 2023.

The cure rate and median length of stay for children who did not share RUTF were 23 (27.4%) and 55 ± 1 days, respectively, while children with shared RUTF were 240 (89.6%) and 81 ± 3 days, respectively [(log-rank test = 60, *p* < 0.001; as shown in Figure 6)].

### Determinants of time to recovery for children with SAM enrolled in the OTP

Candidate independent predictors for the final model in the Cox Proportional-Hazards Model analysis with a *p*-value less than 0.25 were maternal age, resident, sex of index child, maternal educational status, travel time to OTP site, child referral system from their living area, admission criteria, screened by health workers or health extension workers, MAM treatment history, SAM treatment history, diarrhea, cough, amoxicillin, sharing RUTF, selling RUTF, weekly home visit, number of VAS capsules, food security status, child age, delivery place, complementary feeding starting age, child birth order, GMP service utilization frequency, weight gain, and maternal nutritional status were further analysis multiple variable analysis of Cox Proportional-Hazards Model. Family household head, family planning, postnatal care service attendance, maternal occupation, family decision-making for OTP, wealth index, fever, vomiting, and number of under-five children within the household were failed independent variables to be eligible for the multivariable Cox Proportional-Hazards Model with *p*-value<0.25 (Table 7).

**Table 7:** Cox Proportional-Hazards Model multiple variable analyses of determinants of survival/treatment outcomes of OTP in North Wollo Zone, Ethiopia, 2023.

Variable		CHR (95%CI)	AHR (95%CI)	*p*-value
Maternal age in completed years		0.976 (0.957–0.995)	1.012 (0.989–1.037)	0.302
Resident	Urban	0.187 (0.115–0.302)	0.914 (0.483–1.728)	0.781
	Rural	1	1	
Index child sex	Female	2.235 (1.731–2.885)	1.158 (0.877–1.530)	0.301
	Male	1	1	
Maternal education	Illiterate	1	1	
	Read and write	1.994 (1.449–2.743)	1.223 (0.871–1.735)	0.241
	Formal	2.216 (1.621–3.030)	1.497 (1.052–2.130)	0.025^⁎^
Travel time to OTP is an hour	Two and above	0.127 (0.075–0.214)	1.153 (0.585–2.273)	0.680
	Below two	1	1	
Child referral to OTP	Volunteer	1	1	
	Health workers	1.454 (0.540–3.920)	0.705 (0.224–2.221)	0.550
	Self	3.817 (1.796–8.111)	0.782 (0.317–1.931)	0.594
Admission criteria	MUAC	4.123 (2.518–6.750)	1.272 (0.713–2.269)	0.415
	Nutritional edema	1	1	
Screened by	Health workers	0.518 (0.379–0 0.708)	1.330 (0.397–4.455)	0.644
	HEWs	1	1	
MAM treatment history	No	1	1	
	Yes	0.523 (0.384–0 0.714)	0.718 (0.230–2.242)	0.569
SAM treatment history	Yes	0.418 (0.297–0.590)	0.878 (0.577–1.334)	0.541
	No	1	1	
Diarrhea	Yes	0.283 (0.175–0.458)	0.933 (0.531–1.640)	0.810
	No	1	1	

Cough	Yes	0.976 (0.957–0.995)	0.385 (0.176–0.843)	0.017^*^
	No	1	1	
Amoxicillin intake at admission	No	1	1	
	Yes	10.627 (6.435–7.549)	1.925 (0.967–3.834)	0.062
Sharing RUTF	Yes	0.245 (0.160–0.377)	0.997 (0.572–1.737)	0.991
	No	1	1	
Selling RUTF	Yes	0.746 (0.561–0.993)	0.815 (0.588–1.129)	0.218
	No	1	1	
Weekly home visit	Yes	6.841 (3.908–11.974)	1.056 (0.546–2.044)	0.870
	No	1	1	
Number of VAS capsules	Number of capsules	1.532 (1.389–1.691)	1.092 (0.883–1.350)	0.416
Food security status	Secured	1.469 (1.137–1.898)	0.720 (0.502–1.032)	0.074
	Insecure	1	1	
Child age in completed months	6–11	1	1	
	12–23	2.659 (1.678–4.212)	0.714 (0.375–1.361)	0.306
	24–59	1.267 (0.576–2.787)	0.762 (0.264–2.196)	0.615
Delivery place	Home	1	1	
	Health center	3.600 (2.656–4.879)	1.448 (1.023–2.050)	0.037^*^
	Hospital	3.148 (2.203–4.499)	1.207 (0.803–1.813)	0.366
Starting age to complementary feeding in months	Before 6	1	1	
	Above 6	1.259 (0.782–2.026)	1.302 (0.735–2.308)	0.366
	Exactly at 6	2.117 (1.509–2.970)	1.170 (0.805–1.702)	0.411
Childbirth order grouped	First	1	1	
	Second	0.811 (0.556–1.183)	0.730 (0.483–1.104)	0.136
	Third and above	0.778 (0.567–1.065)	0.733 (0.509–1.057)	0.096
GMP service utilization frequency	Numbers	2.086 (1.833–2.374)	1.622 (1.380–1.907)	0.000^⁎⁎^
Weight gain	Weight per g/kg/days	1.048 (0.995–1.103)	1.031 (0.977–1.089)	0.264
Maternal nutritional status in MUAC	23 and above	2.010 (1.459–2.770)	0.566 (0.094–3.408)	0.535
	Below 23	1	1	

AHR, adjusted hazard ratio; CHR, crude hazard ratio; ^⁎^*p*-value < 0.05; HEWs, health extension workers; GMP, growth monitoring and promotion; MAM, moderate acute malnutrition; SAM, severe acute malnutrition; OTP, outpatient therapeutic program. ^⁎^*P*-value < 0.05, ^⁎⁎^*P*-value < 0.0001. VAS, vitamin A supplementation; RUTF, ready-to-use therapeutic food.

### Cox Proportional-Hazards Model showing predictors of time to recovery

The rate of recovery from SAM among children who had amoxicillin at an admission was 2.05 times higher than that of children who did not have amoxicillin at an admission at any time during the study [AHR 2.050 (95% CI, 1.030–4.082)]. At any time during the study, the rate of recovery from SAM among children who had mothers with formal educational status was 1.4 times higher than those children with children who had illiterate mothers [AHR 1.445 (95% CI, 1.019–2.058)]. The rate of recovery from SAM among children without cough was 64% more protective than those children with cough [AHR 0.363 (95% CI, 0.166–0.798)]. At any time during the study, the rate of recovery from SAM among children who had attended GMP service frequently was 1.623 times higher than children who had not attended GMP service utilization frequently [AHR 1.623 (95% CI, 1.382, 1.907)] (Table 7).

## Discussion

This study evaluated the recovery rates of severely uncomplicated malnourished children aged 6 to 59 months who were handled on an OTP. The rate of recovery was 74.72%. Time to recovery was influenced by maternal illiteracy, cough, GMP service utilization, and failure to take amoxicillin.

The rate of recovery reported in this study was nearly identical to the minimum 75% threshold established by the SPHERE standard (37). However, in this study, the recovery rates of severely uncomplicated malnourished children aged 6–59 months were lower in Nigeria (93.4%) (31), Dire Dawa 79.8% (38), Shebedino (79.6%) (19), Afar (83.2%) (35), and Somali (81.7%) (39).

Previous studies in Ethiopia evaluated the recovery rate in the OTP program sites came up with assorted figures as studies that evaluated OTP care reported varying recovery rates in Wolaita 65% (37), Bebchi zone (54.4%) (40), North Gondar zone (65.5%) (36), Wenago district (70.4%) (41), Gobalfti district (65%) (42), Jimma (45%) (43), Bahi Dar city (51.9%) (44), Tigray region (62%), and Kemba district (68%) treated at OTP sites were relatively lower success rates (45, 46) than this study. This study identified defaulters (20.17%), transfers out (5.11%), and death (0%). This defaulter rate was highest when compared with a study conducted in Southern Ethiopia such as Tigray (13.85%) (47), East Hararghe (9.5%) (48), and Shebedino (3.7%) (19). These discrepancies could be because of a variety of reasons, such as variations in study timing/season, the maturity of the OTP program in the research locations, and differences in the underlying drivers of hunger between localities. Another possible reason for this could be the study period, design, and distance to OTP sites (19, 46, 49).

The mean weight gain rate of 6.65 g/kg/day observed was less than the expected rate based on the SPHERE standard, which recommends a weight gain rate greater than 8 g/kg/day (37). Many studies conducted in Ethiopia (44, 50, 51) and in East Africa (52) consistently documented substandard rates of weight gain among SAM cases managed through the OTP. A study in Southern Ethiopia found 4.2, 4.5, and 4.2 g/kg/day weight gain in nutritional edematous and marasmus cases, respectively (50). Another study from Wolaita was a g/kg/day rate (51).

Overall, the median time to recovery was approximately 8 weeks (56 days). It is within the range of the acceptable minimum international standard (8 weeks) (45, 53), and it is well within the Ethiopian protocol for the management of SAM which allows children to stay under treatment for up to 16 weeks (112 days) (5). In the current study, the median recovery time is 56 days, which is 2-fold higher than 28 days, which was reported by SPHERE recommendation (21, 28). The finding was comparatively higher than the study done by Afar and Shebedino 44 and the 50-day median recovery time, respectively (19, 35, 54). Those children with extended time to recover from SAM had crises at different levels, for families, children, and countries. Children with extended time to recovery begin their lives at a marked disadvantage such as learning difficulties in school, learning less as adults, and facing barriers to participation in their communities. Mothers also devote their full time to SAM children and abandon the rest of the children in the household also increases the vulnerability of other family members to SAM. At the country level, there is a direct cost for the treatment of SAM children who are admitted for a prolonged time at OTP indirect cost is the loss of economic development due to lack of productive human power, direct allocation of money for the treatment of unrecovered children with SAM, and its complication. Children treated in OTP sites have a case fatality of less than 5% (55). As the time to recovery is longer, medical complications occur and treated as inpatients because of severe infections including pneumonia, diarrhea, and sepsis have a reported case fatality of 10 to 40% (56). It is also clear that children with complicated SAM have a high ongoing risk of mortality even after discharge (54, 57, 58).

Children who had consistently attended GMP service utilizations had a recovery rate from SAM that was 1.623 times greater than that of children who had not attended GMP service utilizations. Body weight monitoring can serve as an early warning system when rapid weight gain is a typical indicator of nutritional/health status (59) through optimal utilization of GMP services confirmed by a study conducted in Ethiopia (24, 60, 61). The utilization of GMP participation in community interactions may have altered attitudes, habits, and information necessary to adopt the recommended therapeutic feeding plan and the frequency of optimal complementary feeding (62).

In the current study, maternal literacy was identified as a significant predictor of recovery of children from SAM attended at OTP sites. This evidence was supported by studies such as in Ethiopia such as Shebedino (19) and Pakistan (63). Most previous studies were based on secondary medical records reviews and had not explored socio-demographic data that were not registered in the standard OTP cards. However, the finding was plausible and anticipatable as maternal literacy was likely to be associated with better child feeding (64) and caring practice, adoption of nutritional counseling (49), and superior household economic status. Mothers with low literacy skills and no formal education are more likely to experience poor child feeding (65) though health literacy was not associated with severe wasting in India (66).

The hazard of children 6–59 months who had a cough at the time of admission was associated with a 64% longer recovery time from SAM. This finding is supported by studies conducted in the Southwest (40, 45). This could be due to a loss of appetite, as children who had a cough may not eat as much as children who did not. Furthermore, infections, metabolic disorders, vomiting, and severe dehydration (67) in combination reduced the odds of recovery rate. Systematic reviews and meta-analyses that demonstrated the absence of co-morbidities significantly increase the chance of a successful recovery from SAM may support this conclusion (68–71).

Children who were given amoxicillin as part of their usual medicine regimen upon admission healed faster than those who were not given the antibiotic at admission time. The research backed by studies from a distance revealed Afar (35), Tigray (46), Wolaita (51), and North Gondar (36). However, studies from Kambata (72) showed amoxicillin had no difference in time to recovery among SAM patients. Because they can move across the intestinal wall, these enteric bacteria are typically the cause of systemic infection. They also cause malabsorption of nutrients, failure to eliminate substances excreted in the bile, fatty liver, and intestinal damage, and can cause chronic diarrhea (5). Antibiotics, particularly amoxicillin, have a role in the treatment of children with SAM. Standard therapy regimens in SAM include antibiotics, which significantly reduce morbidity and may aid in recovery and growth. Antibiotic treatment is given to all children with SAM upon diagnosis, regardless of whether they have an overt infection, in both inpatient and outpatient facilities (5, 73). Few studies have directly assessed the prevalence and impact of antibiotic use on SAM resistance development (74). Some retrospective clinical sub-studies discovered that antibiotics routinely administered in geographical areas with high SAM are becoming progressively resistant at the population level (73, 75). Antibiotics are used both therapeutically and preventatively in children with SAM (17).

Nearly one in four mothers/caregivers of SAM children engaged in sharing and selling RUTF. A similar conclusion has also been drawn by earlier studies (19, 43, 53, 76).

### Strengths and limitations of the study

The strength of this study was the use of time-to-event data and a robust method of analysis. Because the study was a prospective longitudinal study, it was feasible to demonstrate a temporal cause-and-effect link for the components studied. As every attempt was taken to ascertain the correct treatment outcome of children through follow-up home visits at community level services, rather than classifying them as unknown, the treatment result indicators were accurately described.

The OTP treatment outcome may have been influenced by seasonal variation as the winter season in the study location is a no/low harvest season. Moreover, the results of RUTF sharing and selling behaviors may have been underestimated due to social desirability bias. The discrepancies in this study’s duration, area, and technique of poor health management may explain the gap in potential explanations.

## Conclusion

A recovery rate of 74.7%, and 56 days (8 weeks) median recovery time were found. Maternal literacy, GMP frequency, institutional delivery, and cough at admission were independent predictors for this study. As a result, there should be a greater emphasis on the importance of girls’ (future mothers’) education and nutrition counseling, particularly the integration of GMP service components into institutional delivery/for girls/women who have received little education on how to improve time to recovery and the success of the outpatient therapeutic program. Healthcare providers should receive refresher training in community-based management of acute malnutrition so they can strictly follow the current national standards. Governments and non-governmental groups ought to band together to increase the rate at which children admitted to OTP recover. Though this study did not analyze service qualities connected to health professional skills, it would be beneficial to perform other studies to evaluate using mixed methods.

## Data availability statement

The datasets presented in this study can be found in online repositories. The names of the repository/repositories and accession number(s) can be found in the article/supplementary material.

## Ethics statement

The studies involving humans were approved by Woldia University Institutional Review Board. The studies were conducted in accordance with the local legislation and institutional requirements. Written informed consent for participation in this study was provided by the participants’ legal guardians/next of kin. Written informed consent was obtained from the individual(s), and minor(s)’ legal guardian/next of kin, for the publication of any potentially identifiable images or data included in this article.

## Author contributions

FF: Conceptualization, Data curation, Formal analysis, Funding acquisition, Investigation, Methodology, Project administration, Resources, Software, Supervision, Validation, Visualization, Writing – original draft, Writing – review & editing. SM: Data curation, Supervision, Visualization, Writing – review & editing. GM: Data curation, Supervision, Visualization, Writing – review & editing.
